# Depression, anxiety, sexual function and quality of life in women with hyperprolactinemia

**DOI:** 10.61622/rbgo/2025rbgo7

**Published:** 2025-04-30

**Authors:** Renan Massao Nakamura, Daniela Angerame Yela, Amanda Carvalho Santos, Beatriz Cipriano Ribas, Pedro Henrique Rosa e Silva, Bianca Netto Motta, Gabriela Pravatta Rezende, Cristina Laguna Benetti-Pinto

**Affiliations:** 1 Universidade Estadual de Campinas Campinas SP Brazil Universidade Estadual de Campinas, Campinas, SP, Brazil.

**Keywords:** Hyperprolactinemia, Quality of life, Anxiety, Depression, Sexual function, Hyperpituitarism, Surveys and questionnaires

## Abstract

**Objective:**

To evaluate anxiety, depression, sexual function and quality of life in women with hyperprolactinemia.

**Methods:**

Cross-sectional study with 80 women divided into two groups: 30 women with hyperprolactinemia (Study Group) followed and treated at the endocrine gynecology outpatient clinic and 50 women without hyperprolactinemia, with regular menstrual cycles (Control Group) followed at the family planning outpatient clinic of the State University of Campinas from June 2021 to October 2022. Sociodemographic characteristics, quality of life (SF-36 Questionnaire), sexual function (Female Sexual Function Index Questionnaire), depression (Beck Depression Inventory) and anxiety (Beck Anxiety Scale) were evaluated in both groups. Categorical variables were described as absolute frequency and percentage; numerical variables as mean and standard deviation. Comparison of numerical variables between two groups was performed by Mann-Whitney test, while categorical were compared by Chi-Square or Fisher's exact tests.

**Results:**

The mean age of women with hyperprolactinemia was 39.6±8.1 years and the Control Group was 31.2±9.5 years (p<0.001). There was no difference in anxiety scores (p=0.66), depression (p=0.08) and general sexual function (p=0.08) in both groups. However, women with hyperprolactinemia had lower scores in the domains of pain and arousal and worse functional capacity than Control Group (p<0.05).

**Conclusion:**

Women with hyperprolactinemia under treatment do not show any impairment in their anxiety, depression and sexual function when compared to women without hyperprolactinemia. However, analysis of quality of life showed that women with hyperprolactinemia have poor functional capacity.

## Introduction

Hyperprolactinemia is a condition characterized by elevated levels of the hormone prolactin and can lead to galactorrhea, amenorrhea and infertility.^(1)^ Prolactin is a hormone secreted by the pituitary gland responsible for lactogenesis. Besides this function, this polypeptide also affects ovulation, reproductive behavior and homeostasis.^(2)^

It is well known that hyperprolactinemia affects fertility in women, mainly due to the inhibition of the hypothalamic-pituitary-gonadal axis. But, sexual desire can also diminish in women with increased serum prolactin levels, due mainly to relevant complaints, associated with decreased lubrication and orgasm.^(2)^ Hyperprolactinemia impairs sexual function, leading to deficient luteal phase of the menstrual cycle, and chronic anovulation, resulting in a negative impact on female reproductive life.^(3)^ Most studies assesses the impact of hyperprolactinemia in men's sexual function, but there is evidence of the correlation between prolactin levels and post orgasmic sexual arousal in both sexes.^(4)^

In addition, these women have an incidence of emotional disorders that is up to three times higher than that of healthy people.^(2,3)^ Among the factors that may contribute to these emotional conditions are personal dissatisfaction related to body image. Studies indicate that symptomatic women have a negative perception related to their own image.^(2-5)^

There is evidence that the impact on these women's lives is relevant, mainly due to anxious and depressive symptoms.^(6)^ Few studies assess and track the mental health and quality of life of hyperprolactinemia-affected women.^(7-9)^ Thus, the present study aims to evaluate anxiety, depression, sexual function and quality of life in women with hyperprolactinemia.

## Methods

A cross-sectional study was conducted with 80 women divided into two groups: 30 women with hyperprolactinemia diagnosis based on symptomatology and elevated serum prolactin (Study Group) followed up at the endocrine gynecology outpatient clinic and 50 women without hyperprolactinemia, with regular menstrual cycles (Control Group) followed up at the family planning outpatient clinic of tertiary hospital from June 2021 to October 2022.

Women in reproductive age between18 and 49 years of age were included, while those using medications for emotional lability treatment, such as antidepressants or anxiolytics, or psychoactive substances such as illicit drugs (marijuana, cocaine, crack, and others) were excluded. Women with cognitive impairment that affects understanding of the instruments, other chronic diseases that could impact quality of life, such as rheumatoid arthritis, heart disease, hematologic diseases like sickle cell anemia, kidney disease, liver disease, or neuropathies such as multiple sclerosis, and psychiatric disorders such as mood disorders, including depression or bipolar disorder, or generalized anxiety disorder in both groups were also excluded. Women were invited to participate spontaneously, respecting the inclusion and exclusion criteria. They were interviewed in a private environment, by a gynecologist trained in applying questionnaires.

The sample size was calculated in order to compare the mean scores of sexual function and depression between the groups of women with and without hyperprolactinemia, with estimates obtained from the literature, setting the significance level at 5% and the power of the sample at 80%.^(2)^ Based on the results, it was estimated that a minimum sample of n=34 women (17 in each group) would be representative to compare the scores of sexual function and depression between the two groups.

The evaluated variables were clinical characteristics of women: age, ethnicity (white or non-white), education (elementary school, high school, and college), occupation (yes - wage earner or no - unemployed, homemaker, and student), partner (yes or no), number of pregnancies, parity, religion (yes or no), smoking (yes or no), previous surgery history (yes or no), comorbidities (yes or no), frequency of sexual activity, etiology of hyperprolactinemia, dopamine agonists use, prolactin value (evaluated through electrochemiluminescence method and expressed in ng/mL), anxiety, depression, sexual function, and quality of life.

To assess quality of life, we used SF-36 questionnaire (Short-Form Health Survey), a multidimensional instrument validated in Brazil consisting of 36 questions grouped into 8 dimensions: functional capacity, physical capacity, pain, general health status, vitality, social aspects, emotional aspects, and mental health. The final score can range on a scale from 0 to 100, where the higher the score, the better the quality of life. Among the 8 dimensions, three correspond to physical aspects (functional capacity, physical capacity, and pain), three to psychological aspects (emotional aspects, social aspects, and mental health), and two are related to both aspects (vitality and general health status).^(10,11)^

Sexual function was assessed by the Female Sexual Function Index (FSFI) questionnaire, which was validated for the Portuguese language. The FSFI consists of 19 questions grouped into 6 domains: desire, arousal, lubrication, orgasm, satisfaction, and pain. Each domain receives a score from 0 to 6, except for desire and satisfaction, with minimum scores of 1.2 and 0.8, respectively. Final scores can range from 2 to 36, being the sum of all specific domains. Sexual dysfunction is characterized by a score of 26.55 or less.^(12,13)^

Depression was assessed using the Beck Depression Inventory (BDI), and anxiety was assessed using the Beck Anxiety Inventory (BAI). The BDI consists of 21 items, including symptoms and attitudes related to sadness, pessimism, feelings of failure, lack of satisfaction, guilt, punishment, self-deprecation, self-accusation, suicidal ideation, crying spells, irritability, social withdrawal, indecisiveness, distorted body image, work inhibition, sleep disturbance, fatigue, loss of appetite, weight loss, worry, and decreased libido. Each item is scored from 0 to 3, and the final score is classified as follows: less than 10 points = no or minimal depression, 10 to 18 points = mild to moderate depression, 19 to 29 points = moderate to severe depression, and 30 to 63 points = severe depression.^(14)^

Anxiety was assessed using the Beck Anxiety Scale, which consists of 21 items scored from 0 to 3 that reflect somatic, affective, and cognitive symptoms of anxiety. These symptoms are commonly associated with anxiety disorders, and participants were asked to rate how much they were affected by each symptom during the past week. The final score ranges from 0 to 63 points, with scores of 0 to 7 indicating minimal anxiety, 8 to 15 indicating mild anxiety, 16 to 25 indicating moderate anxiety, and 26 to 63 indicating severe anxiety.^(14)^

To describe the sample profile according to the variables under study, frequency tables of categorical variables were made with values of absolute frequency (n) and percentage (%), and descriptive statistics of numerical variables with values of mean and standard deviation. To compare the categorical variables between the groups, the Chi-Square or Fisher's exact tests were used. To compare the numerical variables between two groups, the Mann-Whitney test was used. The level of significance adopted was 5%. The Statistical Analysis System version 9.4 was used to perform these procedures.

All women who participated in the study signed an informed consent form, and the research was approved by the Ethical Institutional Review Board under the number of the Committee Approval: 4.793.296 (CAEE number 46535121.3.0000.5404).

## Results

The mean age of women with hyperprolactinemia was 39.6±8.1 years and in the Control Group was 31.2±9.5 years (p<0.001). There was no difference in ethnicity, parity, and professional activity in both groups. Most of the women with hyperprolactinemia had lower education and more comorbidities than those in the Control Group (p=0.02 and p=0.007 respectively). There was no difference in the frequency of sexual activity per week in both groups (Table 1). Among women with hyperprolactinemia (30 women), 73.4% had tumor etiology (14 women with microadenoma and 8 women with macroadenoma) and 26.6% had idiopatic etiology, 76.7% were using dopamine agonists, and the median of prolactin value was 40.0 ng/mL.

**Table 1:** Clinical and sociodemographic characteristics among women with and without hyperprolactinemia

Variables	Control Group n=50 mean±SD/ %	Hyperprolactinemia n=30 mean±SD/ %	p-value
Age (years)	31.2 ± 9.5	39.6 ± 8.1	<0.001*
Number of pregnancies			0.23**
Zero (%)	36.0	23.3	
>1 (%)	74.0	76.7	
Ethnicity (white)	42.0	53.3	0.32**
Occupation (yes)	64.0	56.6	0.10***
Partner (yes)	42.0	70.0	0.01**
Education			0.02***
Elementary school	20.0	50.0	
High school	38.0	33.3	
College	42.0	16.7	
Religion (yes)	76.0	100.0	<0.001***
Smoking (yes)	12.0	6.7	0.70***
Previous Surgery History (yes)	6.0	23.3	0.03***
Comorbidities (yes)	28.0	58.6	0.007**
Sexual activity frequency (weekly)	2.3 ± 1.3	1.7 ± 1.6	0.08*

SD: Standard deviation;

* Mann-Whitney test;

** Chi-Square

*** Fisher's exact tests

There was no difference in anxiety scores (p=0.66) and depression scores (p=0.08) in both groups. Most women in both groups had mild depression and minimal anxiety (Table 2). The analysis of quality of life showed that women in the Control Group had better functional capacity than those with hyperprolactinemia (p=0.01). There was no difference in the other domains of quality of life in both groups (Table 3).

**Table 2:** Anxiety and depression among women with and without hyperprolactinemia (n=80)

Variables		Control Group n=50	Hyperprolactinemia n=30	p-value
Depression (%)				
	0-7	34.0	40.0	0.08*
	8-15	36.0	50.0	
	16-25	28.0	6.7	
	>26	2.0	3.3	
Total Score (mean±SD)		11.1±7.8	9.9±8.5	0.39**
Anxiety (%)				0.66*
	0-7	40.0	46.7	
	8-15	24.0	23.3	
	16-25	24.0	13.3	
	>26	12.0	16.7	
Total Score (mean±SD)		13.3±11.4	12.0±10.4	0.69**

SD: Standard Deviation;

* Fisher test;

** Mann-Whitney test

**Table 3:** Quality of life and sexual function among women with and without hyperprolactinemia (n=80)

	Control Group n=50 Mean±SD	Hyperprolactinemia n=30 Mean±SD	p-value*
SF 36			
General Health Status	57.7±17.5	56.4±12.3	0.55
Social Aspects	63.2±25.6	68.7±28.7	0.32
Vitality	48.7±24.2	53.8±21.7	0.38
Mental Health	55.8±20.2	58.4±22.5	0.44
Pain	66.0±21.4	60.0±20.9	0.24
Emotional Aspects	47.3±45.7	54.4±46.7	0.57
Physical capacity	64.5±36.8	60.8±43.8	0.84
Functional Capacity	86.6±16.7	71.0±29.9	0.01
FSFI			
Desire	4.0±1.1	3.8±1.3	0.44
Arousal	4.3±1.7	3.3±1.8	0.007
Lubrication	4.6±1.6	3.8±2.1	0.18
Orgasm	4.2±1.6	3.6±2.1	0.35
Satisfaction	4.6±1.3	4.3±1.7	0.57
Pain	4.6±1.9	3.6±2.3	0.02
Total	26.5±7.6	22.7±9.5	0.08

SD: Standard Deviation; FSFI: Female Sexual Function Index;

* Mann-Whitney test

The Control Group had normal sexual function (26.5±7.6), while women with hyperprolactinemia had sexual dysfunction (22.7±9.5), but there was no significant difference in scores (p=0.08). Women in the hyperprolactinemia group had lower scores in the domains of pain (p=0.02) and arousal (p=0.007) when compared to the Control Group (Table 3).

## Discussion

Women with hyperprolactinemia were older, had more comorbidities, and lower educational levels than women in the Control Group. There were no differences in anxiety, depression, quality of life, and sexual function between the groups, although women with hyperprolactinemia had worse pain and arousal scores than control women.

During the last decades, quality of life has been recognized as an important and comprehensive outcome measure for the efficacy of pharmacotherapy and psychosocial interventions. Similar to our results, a Chinese study also found no difference in quality of life among women with hyperprolactinemia compared with controls.^(15)^

Another study with 55 women with microadenoma-induced hyperprolactinemia treated with dopamine agonists also showed no difference in the quality of life of these women compared to controls, although in this study, women with hyperprolactinemia had worse scores for anxiety and depression than control women.^(6)^

Most studies on the results of hyperprolactinemia treatment focus on clinical and biochemical response rather than functional and emotional well-being. Women with hyperprolactinemia have impaired quality of life due to emotional disorders and fatigue, with increased anxiety and depressive feelings. Therefore, treatment and follow-up should not only focus on biochemical response and recovery of the menstrual cycle but also on persistent psychological impairment.^(6)^

A study evaluating the quality of life of women with pituitary macroadenoma in comparison with women without that diagnosis presented impaired quality of life compared to controls.^(17)^ In general, pituitary diseases are associated with impaired quality of life.^(7)^ This can be explained by various factors. Macroadenomas are associated with different degrees of hypopituitarism, which require hormone replacement. However, despite optimal endocrine replacement strategies, hypopituitarism is associated with impaired quality of life parameters.^(17-19)^ A systematic review of quality of life in pituitary adenomas showed that this disease negatively impacts quality of life, but there is a need for specific questionnaires for prolactinoma and non-functioning adenoma and that psychosocial interventions and appropriate medical treatment can improve quality of life.^(8)^

A Brazilian study with women with hyperprolactinemia due to pituitary microadenoma showed that quality of life is impaired in women with microprolactinoma treated with dopamine agonists. Quality of life was negatively correlated with prolactin levels. Thus, adequate disease control can prevent adverse consequences of hyperprolactinemia on quality of life.^(9)^ In our study, most causes of hyperprolactinemia were due to pituitary tumors, and most women had controlled disease.

In previous studies, women already had impairment over several domains of sexual functions, maintaining the hypothesis that hyperprolactinemia could compromise the quality of the sexual act, affecting lubrication to degree of pleasure or excitement and contributing to pain in the sexual act.^(20,21)^ These data are similar to our results. Among the main complaints of women noted in the questionnaires, the lack of excitement and pain interfered with the quality of sexual life.

It is well established that prolactin can alter hypothalamus-pituitary-ovary axis due to their interaction. Overall, the rise of prolactin affects negatively the secretion of FSH and LH, eventually leading to hypogonadism. This affects not only the fertility of the women,^(4)^ but also leads to vaginal dryness and pain during sexual intercourse. Prolactin is also physiologically linked to orgasm and pleasure during sexual intercourse, considering that areas of the brain related to orgasm show prolactin receptors.^(22)^ So, in women with sexual dysfunction, related to amenorrhea or galactorrhea, a prolactin dosage can be an important ally to an easy and feasible treatment to sexual impairment.^(22)^ Our study has shown decreased arousal and increased pain in sexual intercourse, being aligned to what the literature points.

One study shows that women with hyperprolactinemia not treated with dopaminergic agonists have impaired sexual function when compared to healthy women.^(2)^ The literature shows that cabergoline is a drug that reduces prolactin levels and increases all parameters of sexual desire, function and positive perception of the refractory period.^(23)^

This study has some limitations, the fact that it is not a cross-sectional study that allows conclusions of cause and effect and also that it did not match the population compared by age. Another limitation is that the groups are not homogeneous. There is importance in the difference between the age groups monitored at the outpatient clinic, with an extensive range of variable ages. It is known that the responses of the different age groups to the applied questionnaires can be different, being necessary to enlarge the sample group for comparison between the women themselves followed up due to the hyperprolactinemia condition.

We reaffirm the importance and relevance of a topic that has few evidence, with impacts already demonstrated in other studies on the quality of life of women. Complaints are diverse, with impacts on different issues about women's overall health. In the face of such a relevant and prevalent pathology, with impacts already demonstrated in the literature, we consider it important to continue the evaluation, comparing among women with hyperprolactinemia whether there are differences between women who are already adequately treated and controlled and women without treatment at diagnosis.

Data from the literature show that the psychological repercussions in women with hyperprolactinemia, including depression and anxiety are scarce. Most of our women had hyperprolactinemia due to tumor etiology, which can have a greater impact on psychological aspects.

Women were investigated in a cross-sectional study, which made it impossible to draw cause and effect conclusions. Another limitation may be the difference between the groups as they were not matched by age.

## Conclusion

Women with hyperprolactinemia under treatment do not show any impairment in their quality of life, anxiety and depression leves, when compared to women without hyperprolactinemia. However, regarding sexual function, women with hyperpolactinemia present potential risks of dysfunction, since they had lower scores in the domains of pain and arousal.
